# *GSTM1* Gene Expression Correlates to Leiomyoma Volume Regression in Response to Mifepristone Treatment

**DOI:** 10.1371/journal.pone.0080114

**Published:** 2013-12-04

**Authors:** Mikael Engman, Suby Varghese, Kristina Lagerstedt Robinson, Helena Malmgren, Anna Hammarsjö, Birgitta Byström, Parameswaran Grace L Lalitkumar, Kristina Gemzell-Danielsson

**Affiliations:** 1 Department of Women's and Children's Health, Division of Obstetrics and Gynecology, Karolinska Instituet, Karolinska University Hospital, Stockholm, Sweden; 2 Department of Clinical Genetics, Karolinska University Hospital, Stockholm, Sweden; Clermont Université, France

## Abstract

Progesterone receptor modulators, such as mifepristone are useful and well tolerated in reducing leiomyoma volume although with large individual variation. The objective of this study was to investigate the molecular basis for the observed leiomyoma volume reduction, in response to mifepristone treatment and explore a possible molecular marker for the selective usage of mifepristone in leiomyoma patients. Premenopausal women (N = 14) were treated with mifepristone 50 mg, every other day for 12 weeks prior to surgery. Women were arbitrarily sub-grouped as good (N = 4), poor (N = 4) responders to treatment or intermediate respondents (N = 3). Total RNA was extracted from leiomyoma tissue, after surgical removal of the tumour and the differential expression of genes were analysed by microarray. The results were analysed using Ingenuity Pathway Analysis software. The glutathione pathway was the most significantly altered canonical pathway in which the glutathione-s transferase mu 1 (*GSTM1*) gene was significantly over expressed (+8.03 folds) among the good responders compared to non responders. This was further confirmed by Real time PCR (p = 0.024). Correlation of immunoreactive scores (IRS) for GSTM1 accumulation in leiomyoma tissue was seen with base line volume change of leiomyoma R = −0.8 (p = 0.011). Furthermore the accumulation of protein GSTM1 analysed by Western Blot correlated significantly with the percentual leiomyoma volume change R = −0.82 (p = 0.004). Deletion of the *GSTM1* gene in leiomyoma biopsies was found in 50% of the mifepristone treated cases, with lower presence of the GSTM1 protein. The findings support a significant role for GSTM1 in leiomyoma volume reduction induced by mifepristone and explain the observed individual variation in this response. Furthermore the finding could be useful to further explore GSTM1 as a biomarker for tailoring medical treatment of uterine leiomyomas for optimizing the response to treatment.

**Clinical Trials identifier:**

www.clinicaltrials.gov: NCT00579475, Protocol date: November 2004. http://clinicaltrials.gov/ct2/show/NCT00579475

## Introduction

Uterine leiomyoma is the most frequently reported tumour among women. The highest incidence is seen during the late reproductive period. It is estimated that the incidence of leiomyoma is 29.7 to 45.6 per 1000 patient-years [1], [2]. In the United States alone, the estimated cost for treating uterine leiomyomas was USD 2.1 billion per year and mainly due to the surgical management of the disease [3]. The main reason for surgery is vaginal bleeding or mechanical discomfort due to the position or size of the tumor. Thus medical management of uterine leiomyomas will be of prime importance to improve the quality of life for women in their reproductive years and reduce the concomital financial burden to society.

Leiomyoma tissue overexpress progesterone receptors in comparision to adjacent myometrium and are involved in the process of leiomyoma growth [4]. Thus, the use of selective progesterone receptor modulators (SPRMs) for leiomyoma treatment has been explored and previously addressed in fourteen clinical studies, among which only two were placebo controlled [5], [6]. Recently, treatment with the SPRM ulipristal acetate (UPA) has shown promising results very similar to those reported for mifepristone [7], [8]. SPRMs have shown to be effective for reduction of leiomyoma volume and the associated symptoms [9], [10] with a significant and immediate reduction in vaginal bleeding and increase in hemoglobin levels [6], [11]. However, the mechanisms of action of SPRMs responsible for the observed leiomyoma volume reduction is not completely understood. Moreover, in contrast to the effect on uterine bleeding leiomyoma volume reduction induced by mifepristone showed marked individual variation in response to treatment and was not associated with any change in uterine blood flow [6]. Thus we conducted the present study to further explore the molecular mechanisms responsible for the observed volume reduction in leiomyoma, in response to mifepristone treatment, with the aim to identify potential molecular markers that could be used for screening and identification of leiomyomas suitable for pharmacological management Our results demonstrates that the response to mifepristone with regard to leiomyoma volume reduction correlated to expression of glutathione-s transferase mu 1 (GSTM1). The identification of this potential biomarker could help in improving the response to SPRM treatment.

## Materials and Methods

### Ethics statement

Ethical approval was obtained from the local ethics comittee at Karolinska Institutet. Patients were informed about the study and written consent was obtained from each participant before inclusion in the study and any study related activity. The samples for this study was obtained as part of a clinical trial (Clinical Trials identifier: www.clinicaltrials.gov: NCT00579475), which was published elsewhere (6). The protocol for this trial and supporting CONSORT checklist are available as supporting information; see Checklist S1 and Protocol S1.

### Patient treatment and selection criteria

**Figure 1:**
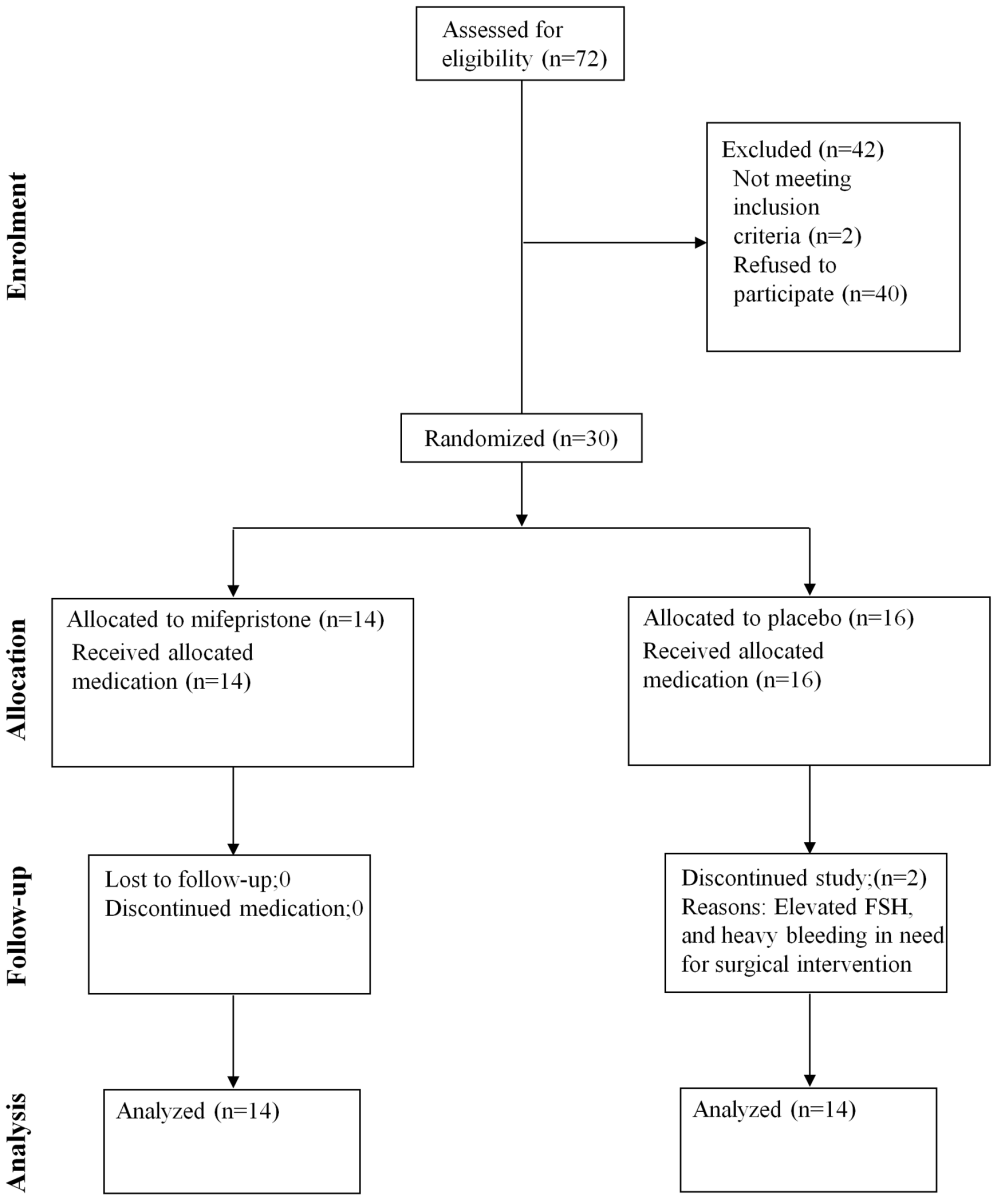
Flow chart explaining the patient enrolment, allotment and relevent details, as per consolidated standards of reporting trials (CONSORT) in the study where leiomyoma patients were treated with mifepristone/placebo.

Women were recruited between 2004 and 2007 for a prospective, double blind, randomised controlled trial of mifepristone, 50 mg on alternate days (Mifegyne®, Exelgyn, France), versus an inactive comparator (Trio-Be®, Recip Sweden) for preoperative treatment of uterine leiomyomas. The treatment duration was 12 weeks and this study was performed at the Department for Women's and Children's Health, Karolinska Institutet and Karolinska University Hospital in Stockholm, Sweden. Premenoausal women with uterine leiomyoma requiring surgical treatment and with no contraindications to mifepristone were included (n = 30). Out of these, 16 women received inactive treatment and the rest 14 patients received mifepristone (fig. 1). Exclusion criteria were hormonal therapy within three months prior to initiation of study medication, intercurrent disease, suspicion of malignancy or need for surgery without further delay. The size of the dominant leiomyoma was monitored ultrasonographically with a vaginal probe, Voluson730 Expert equipment (General Electric, Zipt, Austria) every fourth week. All examinations were done or supervised by the same investigator and the clinical data is published elsewhere (6). Inorder to adress the molecular basis for the observed pronounced variation in response with mifeprstone, in leiomyoma volume reduction, we investigated the differential gene expression and protein accumulation between two extreme subsets of patients, categorised as good or poor responders, as well as for the intermediate response cases. The cut off arbitrary limit was defined as at least 30% reduction of myoma volume as indicator for good response.

### Extraction of total RNA

At surgery, biopsies were obtained from the periphery of the dominant leiomyoma, using a 5 mm dermal punch biopsy tool. The specimens were snap frozen and stored in liquid nitrogen until further homogenization and isolation of total RNA. The leiomyoma samples were homogenized in a Retsch™ tissue mill (Retsch KG, Hahn, Germany) at a frequency of 30/sec for 2 min repetitively with intermittent freezing in liquid nitrogen until the tissue was completely powdered. Total RNA was isolated from the homogenate using Trizol method (Invitrogen). The total RNA was dissolved in RNAse free water and the RNA content was measured using Nanodrop™ 1000 spectrophotometer (Thermo scientific Waltham, MA, USA). The 260/280 ratio was >1.8. The samples were stored at −70°C until further used.

### Microarray

Eight micrograms of total RNA per subject was used in the standard protocol from Affymetrix to label targets. The RNA was reverse transcribed into cDNA, *in vitro* transcribed into labelled cRNA, and hybridized to GeneChip® Human Gene 1.0 ST Array GeneChips (Affymetrix, Santa Clara, CA) according to the Affymetrix GeneChip expression analysis manual. Genes with a change in expression profile of more than 1.5 fold and a statistical significance of p<0.05 were uploaded into Ingenuity Pathway Analysis (http://www.ingenuity.com/) to study the major differences in canonical pathways between the groups.

### cDNA synthesis and Real time PCR

Complementary DNA (cDNA) was reverse transcribed from equal amounts (1 µg) of total RNA after pre-treating with DNAse, using Superscript II™ Reverse Transcriptase (Invitrogen CA USA) and Ribonuclease Inhibitor (Promega Corp., Masden, WI). Real time PCR was performed to determine the relative amounts of each transcript using TaqMan® Gene Expression Assays (Applied Biosystems) and 7300 Real Time PCR System (Applied Biosystems) detection system. Nine probes for the target genes, mainly genes in the glutathione pathway, as identified by microarray, were purchased from Applied Biosystems. The targeted genes chosen were glutathione-s-transferase mu 1 (*GSTM1*), glutathione-s-transferase mu 3 (*GSTM3*), glutathione-s-transferase mu 5 (*GSTM5*), glutathione peroxidase2 (G*PX2*), progesterone receptor (*PGR*), estrogen-receptor alfa (*ESR1*), p21 activated kinase3 (*PAK3*), the proliferation marker *MKI67* and the apoptosis marker *TP53*. Samples were run in triplicates along with proper controls to ensure data validity. The cycling conditions were: 50°C for 2 min, followed by 95°C for 10 min, then 40 cycles of 95°C for 15 s and 60°C for 1 min. After confirming the functionality of the primers using standard samples, cDNA samples from four good 3 intermediate and 4 poor responders to mifepristone treatment were subjected to RT-PCR amplification with the target gene primers. All plates included 18S ribosomal RNA amplification of each sample as an endogenous control for data normalization. The data were analyzed using comparative Ct method, where Ct is the cycle number at which the fluorescence first exceeds the threshold. The relative fold change (**Δ**Ct) was calculated by subtracting the value of the endogenous control from the Ct value of the target gene. The mean ΔCts for the poor responders was then subtracted from the mean **Δ**Cts in the good responder group providing a **ΔΔ**Ct value. By applying the formula 2^−**ΔΔ**Ct^ the fold changes of gene expressions could be calculated [12].

### Immunohistochemical analysis

Accumulation of GSTM1 in the leiomyoma biopsies was studied by immunohistochemistry. The tissue sections were deparaffinized and treated with hydrogen peroxide to inactivate tissue peroxidase activity. Following rinse in TBS, sections were incubated with primary antibody over night at 4°C. After incubating with corresponding probe and polymer horseradish peroxidase molecule (Biocare, USA) and rinsing in TBS, followed by DAB incubation. A negative control excluding the primary antibody was included in the experiment. After light counterstaining with haematoxylin, the sections were evaluated by two independent blinded investigators using the IRS (ImmunoReactiveScoring) system – a semi-quantitative subjective scoring system based on both distribution and intensity of the staining as follows. We applied a multiplicative modified “Quick”scoring with the staining intensity 0–3 (no staining, weak, intermediate or strong staining) multiplied with a score for percentage (1–4) between <25 (1), <50 (2), <75 (3), <100 (4)% of myoma cells and matrix stained. The mean IRS of four microscopic ×200 magnification fields was used for scoring of the specimens.

### Western blot analysis

Tissue lysates were prepared from leiomyoma biopsies of good (N = 4), intermediate (N = 3) and poor (N = 4) responders, in ice cold RIPA buffer (50 mM Tris-HCl, pH 7.5, 150 mM NaCl, 1% NP 40, 0.5% sodium deoxycholate, 10 mM phenyl methyl sulfonyl fluoride, 1 µg/ml of aprotinin, 100 mM EGTA, 100 mM sodium orthovanadate and 100 mM DTT by homogenizing the tissue in a Retsch mill. Tissue lysate extracts (40 µg protein) were resolved on a 4–12% NuPAGE Bis-tris gel. After gel electrophoresis, the proteins were transferred to a PVDF membrane, blocked with blocking buffer for 45 minutes, probed with corresponding antibodies to GSTM1 (AP6896b, Abgent) and *β* actin (A5441, Sigma) and complementary IRDye secondary antibodies. The band was detected and quantified using Odyssey infrared imaging system. The band intensity was quantitated using *β-*actin as endogenous control. The GSTM1 protein accumulation was normalized to *β*-actin as an accumulation quotient for quantitative comparison of the respective samples.

### Multiplex Ligation-dependent Probe Amplification (MLPA) of GSTM1 gene

Multiplex ligation-dependent probe amplification (MLPA) analysis was performed using an available probe set with two independent probes for the *GSTM1* gene (SALSA MLPA kit P128 from MRC-Holland, Amsterdam, the Netherlands). MLPA was carried out according to the provider's recommendations, with the exception that the PCR-reactions were performed in a 25 µl reaction volume. Amplification products were identified and quantified by capillary electrophoresis on an ABI 3500×l Genetic Analyzer (Applied Biosystems) and the accompanying software. The tracing data was imported into and analyzed in Gene Marker software v1.7 (Soft Genetics LLC, State College, PA). The normalized quotients for the different probes were considered as a homozygous deletion when absent in the resulting output.

### TUNEL assay (Terminal deoxynucleotidyl transferase dUTP Nick End Labeling assay)

For assessment of the degree of apoptosis *in situ* end labeling of the 3′OH end of the DNA fragmentation was performed using the “*In Situ* Cell Death Detection kit, Fluorescein” (Roche Applied Science), as per the manufacturer's protocol. Paraffin embedded leiomyoma tissue sections were dewaxed by heating the slides for 1 hour. By serial washings in xylene, a graded series of alcohol and finally distilled water, the tissue was rehydrated. The tissue sections were permeabilised by treatment with proteinase K. The sections were then incubated with the TUNEL reaction mixture (TUNEL labeling solution and enzyme) for 1 h at 37°C in the dark. The slides were then rinsed with phosphate-buffered saline thrice and counter stained with DAPI (4′. 6-diamidino-2-phenylindole) and mounted with antifade for optimal maintenance of fluorescence. Positive controls were prepared by treating slides with DNAse I (1000 U/ml) for 10 min at 25°C before incubating with TUNEL reaction mix. Negative controls were prepared by incubating the slide with TUNEL labeling solution without enzyme. Analysis and scoring under a fluorescence microscope with excitation wavelength 450 nm was performed by two independent investigators. The mean of 4 scores, apoptotic index (AI), was calculated for each case and statistically analysed. The correlation, assessed for 18 inter individual scores, was good, R = 0.8 (p = 0.0002).

### Statistical analysis

The significance analysis of microarrays (SAM) method was used as a statistical approach to analyze the data, focusing on changes in gene expression identified by MAS 5.0. SAM calculates a score for each gene on the basis of the change in expression relative to the standard deviation of all measurements. For genes with scores greater than an adjustable threshold, SAM uses permutations of the repeated measurements to estimate the percentage of genes identified by chance, i.e., the false discovery rate. The *q* value for each gene represents the probability that it is falsely classified as significantly changed. Processing in Ingenuity Pathway Analysis® utilises the Fischer's exact test. Analysis for group differences in gene expressions (**Δ**Cts) were performed using nonparametric methods such as Mann Whitney-U test and Fisher contingence table analysis in the case of GSTM1 since expression or no expression would be more correct than Ct values, as the control group do not express this gene at all. Spearman rank analysis test was used for non parametric analysis of correlations.

## Results

At the end of treatment, a median volume reduction of −23% (range: −81 to +19%) was observed in the mifepristone group which was significant (p<0.01), compared to the control group. However individual response to mifepristone showed pronounced variation with a significant difference between good (<−30%) and poor responders (>−17%) (p = 0.020. Interestingly, two non-responders showed an increase in leiomyoma volume of 17 and 19% respectively. In order to compare the gene expressions between the good and the poor responders to mifepristone treatment, extreme cases each in the good and poor response category as well as 3 intermediate responders, were selected for further analysis of gene and protein expressions. Thus, the mifepristone treated cases were subgrouped as good responders (N = 4) with median volume change cut off limit of −48%, intermediate (N = 3) with median volume change cut off limit of −22% (range: <−21 to <−30%) and poor responders (N = 4) with median volume change cut off limit of +4%, (range: >−20%).

### Microarray

The mean expression values of genes in poor and good reponders were assessed by microarray and further analysed utilising Ingenuity Pathways Analysis (IPA, Ingenuity® Systems, www.ingenuity.com). Twentyone canonical pathways showed significant difference in expression levels (p<0.05) comparing good with poor responders to mifepristone treatment (Table 1). Overall, 641 genes were up or down regulated and changed significantly (p<0. 05) among 34648 genes worked out by microarray (Microarray data- see Checklist S1). In total 22 genes were up regulated more than 5 fold in the good responder group, whereas 15 were down regulated more than 5 fold. The most differently expressed pathway was Metabolism of Xenobiotics by Cytochrome P450 pathway (p = 1.7*10^−7^) where only UGT2B7 was down regulated and 8 genes namely AKR1C2, AKR1C3, AKR1C4, DHRS9, GSTM1, GSTM2, GSTM5, GSTM3, were up regulated among 209 possible genes.

**Table 1:** Differentially expressed genes, with mifepristone treatment: Genes, their cellular location and the fold changes with mifepristone treatment on leiomyoma patients as analysed by microarray followed by IPA.

Symbol	Entrez Gene ID	Entrez Gene Name	Fold Change	Location	Type(s)	Biomarker Application(s)
**GSTM1**	2944	glutathione S-transferase mu 1	8	Cytoplasm	enzyme	Diagnosis, Efficacy, Prognosis
**GSTM3 (includes EG:294)**	2947	glutathione S-transferase mu 3 (brain)	2.3	Cytoplasm	enzyme	Diagnosis, Disease Progression, Prognosis
**GSTM5**	2949	glutathione S-transferase mu 5	2.2	Cytoplasm	enzyme	Diagnosis, Prognosis
**ALDH1A1**	216	aldehyde dehydrogenase 1 family, member A1	2.1	Cytoplasm	enzyme	Disease Progression
**IL18**	3606	interleukin 18 (interferon-gamma-inducing factor)	1.7	Extracellular Space	cytokine	Efficacy, Unspecified Application
**AKR1C4**	1109	aldo-keto reductase family 1, member C4	1.7	Cytoplasm	enzyme	-
**TLR4**	7099	toll-like receptor 4	1.7	Plasma Membrane	transmembrane receptor	-
**CD86**	942	CD86 molecule	1.7	Plasma Membrane	transmembrane receptor	Efficacy, Prognosis
**TNFAIP3**	7128	tumor necrosis factor, alpha-induced protein 3	1.6	Nucleus	other	-
**DHRS9**	10170	dehydrogenase/reductase (SDR family) member 9	1.6	Cytoplasm	enzyme	-
**SYK**	6850	spleen tyrosine kinase	1.6	Cytoplasm	kinase	-
**NFIB**	4781	nuclear factor I/B	1.6	Nucleus	transcription regulator	-
**TLR1**	7096	toll-like receptor 1	1.6	Plasma Membrane	transmembrane receptor	-
**AKR1C3**	8644	aldo-keto reductase family 1, member C3 (3-alpha hydroxysteroid dehydrogenase, type II)	1.6	Cytoplasm	enzyme	-
**VWF**	7450	von Willebrand factor	1.6	Extracellular Space	other	Diagnosis, Efficacy, Prognosis
**CLEC7A**	64581	C-type lectin domain family 7, member A	1.5	Plasma Membrane	transmembrane receptor	-
**UCP2**	7351	uncoupling protein 2 (mitochondrial, proton carrier)	1.5	Cytoplasm	transporter	-
**GSTM2**	2946	glutathione S-transferase mu 2 (muscle)	1.5	Cytoplasm	enzyme	Diagnosis, Prognosis
**F13B**	2165	coagulation factor XIII, B polypeptide	−1.7	Cytoplasm	enzyme	-
**GPX2**	2877	glutathione peroxidase 2 (gastrointestinal)	−1.7	Cytoplasm	enzyme	Efficacy
**UGT2B7**	7364	UDP glucuronosyltransferase 2 family, polypeptide B7	−2.2	Cytoplasm	enzyme	Efficacy, Prognosis

The second most significant pathway was glutathione pathway, harboring glutathione-s tranferases (GST, p = 6.3*10^−5^, ratio 5%). One of 98 (1%) genes was downregulated (GPX2 – 1.7 fold) and 4/98 genes (4%) were upregulated by good response to mifepristone treatment. GSTM1 (+8.0-fold), GSTM2 (+1.5 fold), GSTM3 (+2.3 fold) and GSTM5 (+2.2 fold) for the good responder group. IPA analysis showed that GSTM1 and GSTM2 are also part of 6 other significantly overexpressed canonical pathways where as GSTM3 and GSTM5 are involved in 5 further pathways out of the 21 significantly altered pathways. Genes in the glutathione pathway are also present in Nrf-2 mediated antioxidant pathway (p = 0.006).

### Real time-PCR

Real Time -PCR amplification of the genes in the glutathione pathway showed a differential expression of the genes belonging to glutathione pathway (fold changes in brackets). GSTM1 was analysed in contingence table and Fisher test as poor and intermediate responders in general had no detectable levels of GSTM1. For GSTM3 (+3.06) and GSTM5 (+1.53) the fold changes were not significant. A set of reference genes namely, ESR1 (+2.04), PGR (−1.12) and PAK3 (+1.31), MKI67 (+1.98) and TP53 (+1.27) were also analysed and did not have significant differences. Interestingly, GSTM1 was not expressed at all among non responders or intermediates. Inclusion of the intermediate cases along with non responders showed a significant difference between good and poor responders (p = 0.024). Expression of glutathione peroxidase (GPX2) was not seen in any case. Estrogen receptor alfa was upregulated 2-fold, for good responders without a significant change. Upregulations of TP53 (+1.27 fold, n.s.) and MKI67 (+1.97 fold n.s.) were seen for good responders, ESR1 gene expression was significantly correlating to the gene expression of TP53, R = 0.88 (p = 0.004). (Table 2)

**Table 2:** Glutathione pathway gene expression and leiomyoma volume reduction: Expression levels and fold changes of differentially expressed genes in glutathione pathway in good and poor responders for leiomyoma patients with 12 weeks of mifepristone treatment as studied by with 12 weeks of mifepristone treatment in leiomyoma tissue as studied by real time PCR and microarray (*The least responder did not have any detectable Ct value for GSTM1).

Gene	Good responders (N = 4)				Poor responders (N = 4)				Fold change for good responders	
	Median*	Min	Max	Quartile	Median	Min	Max	Quartile	RT-PCR	Micro-array
**GSTM1**	19.61	17.34	19.71	2.38	UD	UD	UD	UD	-	+8.03
**GSTM3**	17.22	15.37	18.61	1.99	18.41	18.02	20.06	1.11	+3.06	+2.26
**GSTM5**	17.22	16.65	22.80	3.14	19.22	17.75	20.14	1.65	+1.53	+2.23
**PAK 3**	20.94	20.04	21.46	0.80	21.21	20.58	21.93	1.08	+1.31	−1.01
**PGR**	15.04	14.21	15.85	0.96	15.03	14.15	15.30	0.67	−1.12	+1.09
**ESR1**	13.01	11.91	14.10	1.80	14.05	13.61	14.43	0.52	+2.04	+1.09
**MKI67**	19.94	19.28	23.07	1.98	21.60	20.90	22.08	0.80	+1.98	−1.02
**TP53**	16.61	16.17	17.60	0.96	17.21	16.67	17.30	0.38	+1.27	+1.19

### Immunohistochemical analysis of GSTM1

**Figure 2:**
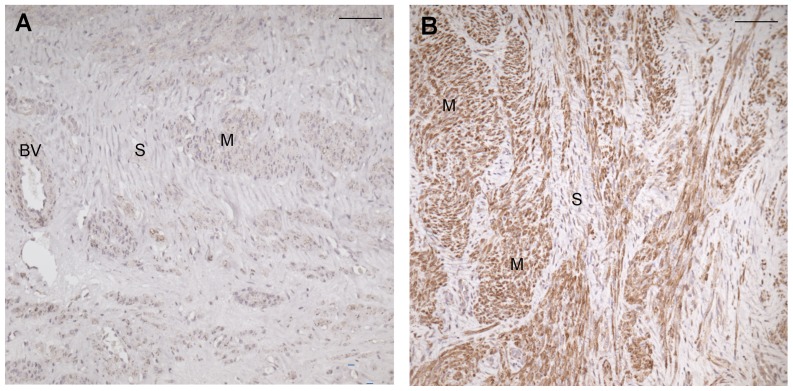
Immunohistochemical analysis of GSTM1 show low immunostaining in poor responder (A) compared with good responder (B) to mifepristone treatment. Muscle bundles (M) in good responders had higher accumaulation of GSTM1 protein than the stroma (S). Poor responders showed very low immunostaining for GSTM1 in muscle budle (M) and the smooth muscle cells of blood vessels (BV). The stroma (S) was almost negative. (Bar = 12 µ).

**Figure 3:**
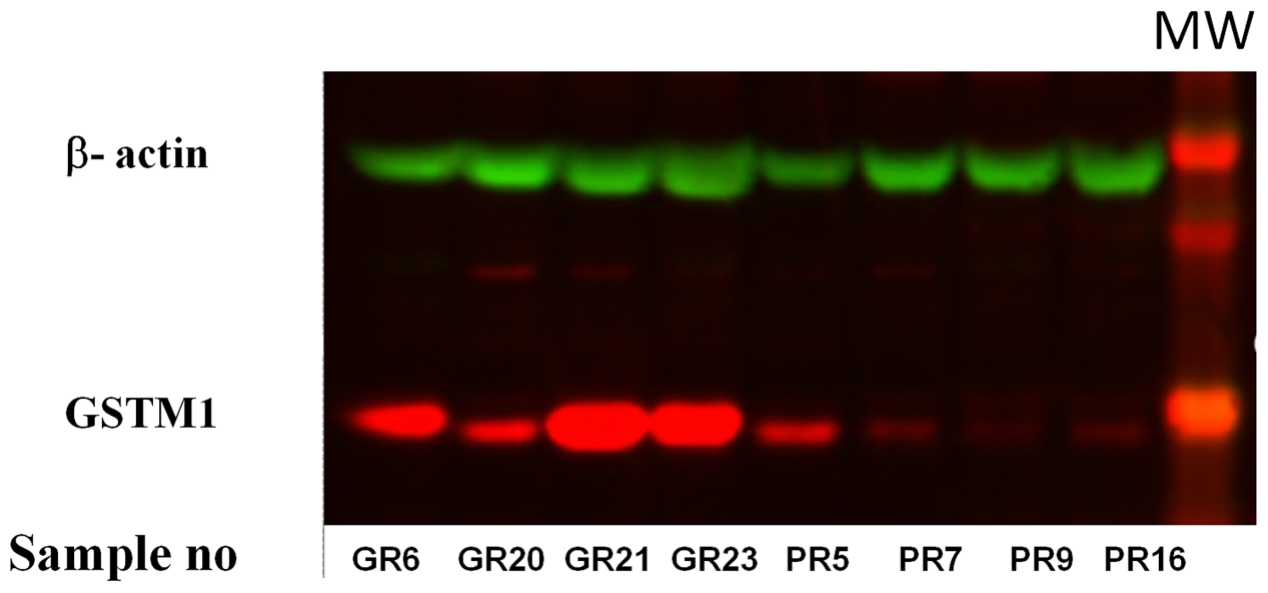
Accumulation of GSTM1 protein in GR (good responders) or PR (poor responders to mifepristone treatment as demonstrated by Western Blot.

Areas with myoma fibromuscular bundles and areas with extracellular matrix were evaluated. The good responder group had median (range) IRS of 5,75 (0.50 to 12) in fibromuscular parts and 3.0 (0.5 to 9) in the matrix (Fig. 2). For poor responders IRS fibromuscular bundle area was 0.25 (0 to 6) (p = 0.114) and for matrix 0.25 (0 to 2.25) (p = 0.057). Significant difference in IRS score between good and poor responders was not seen neither in the bundles nor in the matrix. Pooled IRS_matrix_ values of good and poor response categories showed a negative correlation to leiomyoma volume change (R = −0.83; p = 0.011). Strong correlation was seen for IRS_GSTM1_ in bundles (R = 0.97, p<0.001) and matrix (R = 0.99, p<0.001) versus GSTM1 protein accumulation as analysed by Western blot, categorised according to good or poor response to mifepristone treatment as well as when regarding GSTM1 +/− gene deletion status (Fig. 3).

### Western Blot analysis

The protein accumulation of GSTM1 for good and poor responders to mifepristone, as well as 3 intermediate cases were assessed using Western blot for GSTM1 accumulation normalised to β-actin. GSTM1 were highly expressed in the 3 strongest of the four good responders, GSTM1/beta-actin quotient median was 1.51 (min-max: 0.37–5.12). In contrast low accumulation were generally seen among poor and intermediate responders, the median for normalised values was 0.08 (range: 0.05 to 0.60). However the difference was only close to a statistical significance (p = 0.057). The negative correlation between GSTM1 protein accumulation and leiomyoma volume change was highly significant assessing 10 mifepristone treated cases, R = −0.82 (p = 0.004, Fig. 3).

### Correlation between immunohistochemistry and Western blot

Accumulation of GSTM1 protein, as determined by Western blot, was confirmed to be significantly higher (median 1.51 range: 0.60–5.12, p = 0.047) in the GSTM1^+^ group (N = 4) compared to the GSTM1^0^ cases (N = 7, median 0.08; range 05–0.37). By categorisation according to GSTM1 deletion or not, there were significant differences between categories in IRS_GSTM1_ (N = 8) score both in leiomyoma smooth muscle cell bundles (p = 0.030) and matrix (p = 0.030) of GSTM1^+^. For the GSTM1^0^ cases the IRS scores were in smooth muscle cell bundles was 0.25 (0.0–0.5) and in matrix 0.25 (0.0–0.5). The pooled IRS cases in smooth muscle cell bundles (N = 8) and matrix (N = 8) strongly correlated R = 0.97 (p<0.001), R = 0.99 (p<0.001) to the leiomyoma protein accumulation as assessed by Western blot.

### TUNEL analysis

There was no significant difference in apoptotic index (AI) between good (median 4.2%, min 1.4- max 7.8%) and poor responders (median 5.0%, 4.7%–22%) as evaluated by TUNEL assay. Higher scores with pronounced variation were seen for poor compared to good responders.

### Analysis of GSTM1 deletion in leiomyoma

MLPA analysis of the GSTM1 showed gene deletion in 7 of 14 mifepristone treated cases (50%), and non deletion was present in 5 of 14 cases (36%). Two cases were lacking data for GSTM1 copy number, as it was not possible to obtain sufficient remaining leiomyoma tissue. Data for leiomyoma volume reduction as well as protein accumulation were analysed and categorised according to the expression of GSTM1 gene by MLPA analysis. For the GSTM1^+^ the median volume reduction was −59% (range: −81 to −9) and for the GSTM1^0^, it was −20% (range: −31–+19). The statistical significance in the volume change was, p = 0.05 between the GSTM1^+/0^ categories.

## Discussion

**Figure 4:**
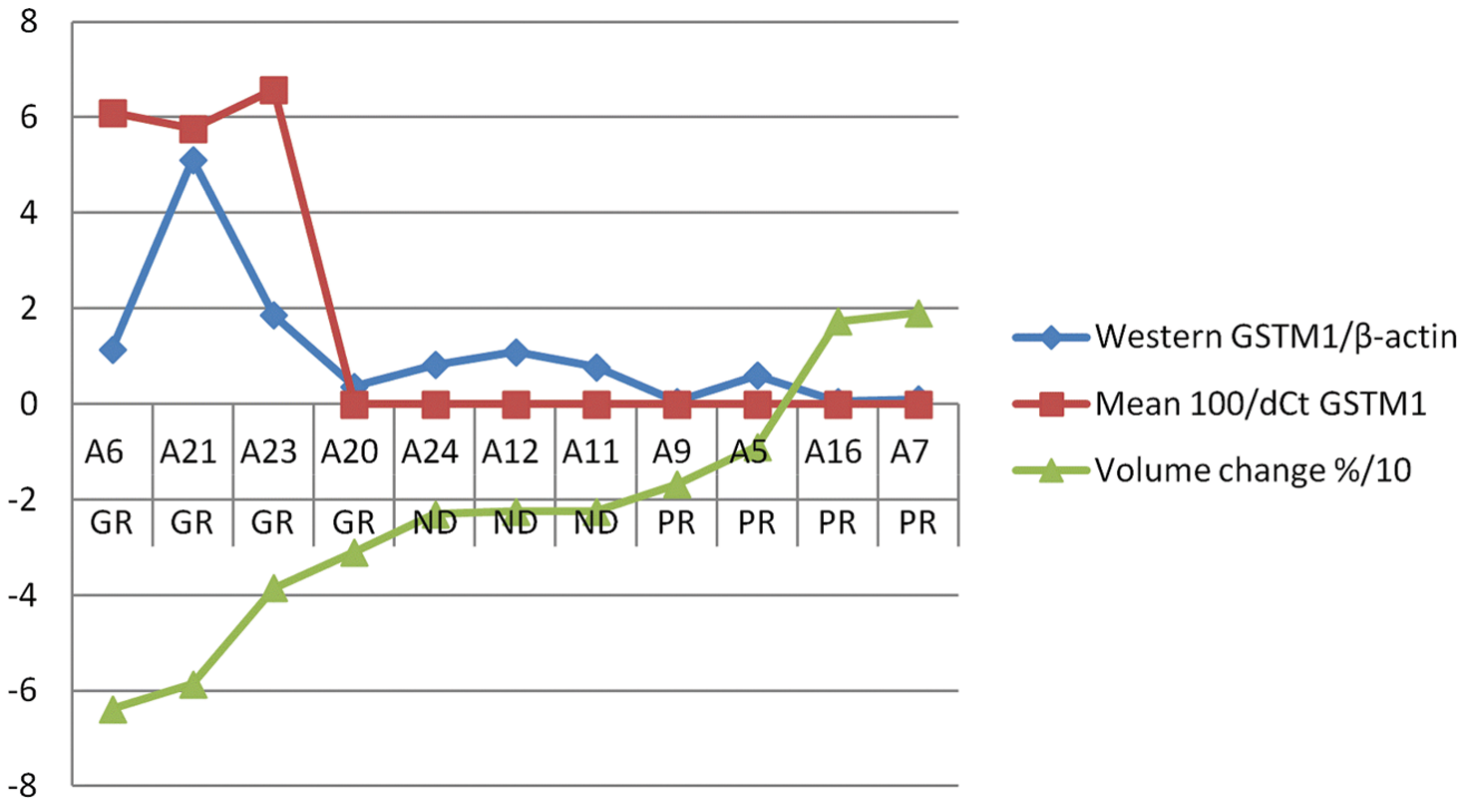
Correlation of leiomyoma volume change (percentage ×0.1) and the expression levels of GSTM1 as studied by real time PCR and Western blot GR = good responders, PR = poor responders, ND = not determined (in between groups) category.

This study was done to explore the molecular pathways involved in the observed individual response in leiyomyoma volume reduction to mifepristone treatment, which was reported earlier [6]. To address this, we looked into the differential expression of genes by microarray, between good and poor responders to mifepristone treatment in terms of volume reduction. Our initial finding from the microarray of the unique expression pattern of GSTM1 in good responders was further reconfirmed using more robust techniques such as real time PCR and immunohistochemistry. Although, with small numbers in each group of this study, we have a consistent and significant result showing that the leiomyoma volume reduction is associated with GSTM1 expression (Fig. 2, 3, 4 and table 3). We, further looked into the polymorphism of GSTM1 and found near close assocaition between the leomyoma volume reduction and its gene polymorhism.

**Table 3:** GSTM 1 expression leiomyoma volume reduction: Comparison of GSTM1 expression as seen by by real time PCR and Western Blot in relation to percentage of leiomyoma volume change during 3 months of mifepristone treatment.

Response	Sample ID	Volume change	WB GSTM1/β-actin	Mean 100/dCt GSTM1
**GR**	6	−63.8	1,15	6,10
**GR**	21	−58.6	5,12	5,77
**GR**	23	−38.5	1,86	6,58
**GR**	20	−30.9	0,37	*
**ND**	24	−23.1	0,83	*
**ND**	12	−22.5	1,09	*
**ND**	11	−22.4	0,78	*
**PR**	9	−16.8	0,05	*
**PR**	5	−08.9	0,60	*
**PR**	16	17.3	0,05	*
**PR**	7	19.1	0,10	*

GR-good responders (>30% volume reduction); PR-poor responders (<18% volume reduction).

* no gene expression detected.

Glutathione-S transferases (GST) or GSTM1 are cytosolic or membrane bound enzymes involved in the metabolism of steroids, reactive oxygen species (ROS), xenobiotics, drugs and carcinogens by formation of thioethers and vascular smooth muscle cell regulation. GSTM1-null variant with prevalence of 41–54% in six different populations is described with a deletion of the *GSTM1* gene [13]. Several studies correlate *GSTM1* deletion with increased risk of cancer [14], [15], [16] indicating is possible antiproliferative activity. Interestingly, susceptibility for endometriosis, which is higly hormone dependent was shown in one study to be negatively correlated to the expression of *GSTM1* gene although this was challenged in other studies [17] [18], [19]. We demonstrated a good correlation in leiomyoma volume reduction in response to mifeprsitone with the expression of GSTM1 in the tissue. Reports show that Glutathione-S tranferases abundance is of importance in the regulation of the cell-cycle either by facilitating apoptotic or inhibiting proliferative cell-cycle associated pathways. The role of GSTM1 in leiomyoma could be related to cell-cycle or metabolic events through regulatory metabolism of the steroid hormone receptor and its ligands. In connection to our finding of 50% gene polymorphism, an earlier report that *GSTM1* deleted phenotype has an association with increased risk for leiomyoma develoment [20]. ROS and NADPH oxidation were recently considered of importance for regulation of cytokine effects on the mitogen activated pathways related to cell proliferation in leiomyoma cell lines [21]. Antioxidant response elements (AREs) upstream glutathione genes are induced by the transcription factor Nrf-2 upon exposure to ROS [22]. In the present study induction of the Nrf-2 pathway was found to be significant. Thus, it seems likely that GSTM1 is involved in cell cycle regulation in part through mediating an anti-oxidative effect of mifepristone. In support of this, studies have shown that mifepristone reduced proliferation of endometrial cell line via the antioxidant effect [23].

A previous study on leiomyoma has reported an increased proliferation with an over-accumulation of MKI-67 in the tissue and that TP53 was unaffected in leiomyoma tissue compared to adjacent myometrium [24]. Also, our previous study on breast tissue showed marked reduction in breast cell proliferation as result of mifepristone treatment [25]. Thus we tested whether leiomyoma volume regression is associated with any reduction in cell proliferation and increased apoptosis using TUNEl assay. Although there was an increased accumulation of this molecule in good responders this did not reach significance. It is possible that a larger sample size might have given a different result.

As previously reported all mifepristone treated patients had almost complete relief of vaginal bleeding which was the clinically most significant finding, while the individual response in regression of leiomyoma volume varied [6]. Leiomyoma volume reduction could be of importance for clinical symptoms such as pain and pressure and furthermore from a surgical management point of view. Good response to medical treatment will avoid the need for expensive surgical intervention, at least in a subset of patients. It is likely that medical treatment of leiomyoma will increase in the near future as Ulipristal acetate, a SPRM has recently been approved for the treatment of leiomyoma in Europe (7).

Based on the available information on GSTM1, [19]. We also see a good correlation of GSTM1 expression with leomyoma volume reduction. Thus, it is worth exploring further with large clinical samples for patient selection, before planning medical management for leiomyoma with SPRMs. We conclude that GSTM1 could be a potential biomarker for predicting or possible optimising the response to SPRMs treatment for the clinical management of uterine leiomyoma, which could save economic burden and impove quality of life for affectedwomen.

## Supporting Information

Checklist S1 **Table explaining the location of background, study design, outcome, results and discussion in the article.**
(DOC)

Protocol S1 **Details of the protocol of the study ‘The effect of preoperative treatment with mifepristone on uterine fibroids and breast tissue’.**
(DOC)
