# Supplementary material for: GSTM1 Gene Expression Correlates to Leiomyoma Volume Regression in Response to Mifepristone Treatment
Source: PLoS One. 2013 Dec 4;8(12):e80114. doi: 10.1371/journal.pone.0080114 (PMC3851176; doi:10.1371/journal.pone.0080114)
Supplement: Checklist S1 — Table explaining the location of background, study design, outcome, results and discussion in the article. (DOC) [file pone.0080114.s001.doc]

| Checklist of items to include when reporting a randomized trial | | | | | |
| --- | --- | --- | --- | --- | --- |
|  | | |  |  | **Location** |
| **Section and Topic** | | | **Item #** | **Descriptor** | **Reported** |
| **Title and Abstract** | | | 1 | How participants were allocated to interventions (eg "random allocation," "randomized" or "randomly assigned") | Abstract |
| **Introduction** | | |  |  | Introduction |
|  | Background | | 2 | Scientific background and explanation of rationale |  |
| Methods | | |  |  | Patient treatment and selection criteria |
|  | Participants | | 3 | Eligibility criteria for participants and the settings and locations where the data were collected |
|  | Interventions | | 4 | Precise details of the interventions intended for each group and how and when they were actually administered | Patient treatment and selection criteria |
|  | Objectives | | 5 | Specific objectives and hypotheses | Introduction |
|  | Outcomes | | 6 | Clearly defined primary and secondary outcome measures and, when applicable, any methods used to enhance the quality of measurements (eg multiple observations, training of assessors) | Introduction  Results |
|  | Sample Size | | 7 | How sample size was determined and, when applicable, explanation of any interim analyses and stopping rules | Patient treatment and selection criteria |
|  | Randomization | |  |  |  |
|  |  | Sequence generation | 8 | Method used to generate the random allocation sequence, including details of any restriction (eg blocking, stratification) | Patient treatment and selection criteria |
|  |  | Allocation concealment | 9 | Method used to implement the random allocation sequence (eg numbered containers or central telephone), clarifying whether the sequence was concealed until interventions were assigned |
|  |  | Implementation | 10 | Who generated the allocation sequence, who enrolled the participants, and who assigned participants to their groups |
|  | Blinding (masking) | | 11 | Whether or not participants, those administering the interventions, and those assessing the outcomes were blinded to group assignment. If done, how the success of blinding was evaluated | Patient treatment and selection criteria |
|  | Statistical methods | | 12 | Statistical methods used to compare groups for primary outcome(s); methods for additional analyses, such as subgroup analyses and adjusted analyses | Statistical analysis |
| Results | | |  |  |  |
|  | Participant flow | | 13 | Flow of participants through each stage (a diagram is strongly recommended). Specifically, for each group report the numbers of participants randomly assigned, receiving intended treatment, completing the study protocol, and analyzed for the primary outcome. Describe protocol deviations from study as planned, together with reasons | Fig.1 |
|  | Recruitment | | 14 | Dates defining the periods of recruitment and follow-up | Patient treatment and selection criteria |
|  | Baseline data | | 15 | Baseline demographic and clinical characteristics of each group | Patient treatment and selection criteria |
|  | Numbers analyzed | | 16 | Number of participants (denominator) in each group included in each analysis and whether the analysis was by "intention-to-treat." State the results in absolute numbers when feasible (eg, 10/20, not 50%) | Fig 1 |
|  | Outcomes and estimation | | 17 | For each primary and secondary outcome, a summary of results for each group, and the estimated effect size and its precision (eg 95% confidence interval) | Ref 6 |
|  | Ancillary analyses | | 18 | Address multiplicity by reporting any other analyses performed, including subgroup analyses and adjusted analyses, indicating those prespecified and those exploratory | Materials and methods |
|  | Adverse events | | 19 | All important adverse events or side effects in each intervention group | NA |
| Discussion | | |  |  |  |
|  | Interpretation | | 20 | Interpretation of the results, taking into account study hypotheses, sources or potential bias or imprecision, and the dangers associated with multiplicity of analyses and outcomes | Discussion |
|  | Generalizability | | 21 | Gereralizability (external validity) of the trial findings | NA |
|  | Overall evidence | | 22 | General interpretation of the results in the context of current evidence | Discussion |

This checklist is found at www.consort-statement.org/
