# Supplementary material for: GSTM1 Gene Expression Correlates to Leiomyoma Volume Regression in Response to Mifepristone Treatment
Source: PLoS One. 2013 Dec 4;8(12):e80114. doi: 10.1371/journal.pone.0080114 (PMC3851176; doi:10.1371/journal.pone.0080114)
Supplement: Protocol S1 — Details of the protocol of the study ‘The effect of preoperative treatment with mifepristone on uterine fibroids and breast tissue’. (DOC) [file pone.0080114.s002.doc]

# **Project title:**

# **The effect of preoperative treatment with mifepristone on uterine fibroids and breast tissue**

**Principal investigator:**

Kristina Gemzell Danielsson MD PhD

**Address:**

Department of Woman and Child Health

Division for Obstetrics and Gynecology

Karolinska Hospital/ Institute

S-171 76 Stockholm, Sweden

Telephone: +46 8 5177 2128

Fax: +46 8 5177 4314

E-mail: kristina.gemzell@kbh.ki.se

**Centre:**

WHO collaborating Centre for Research in Human Reproduction

Kvinnokliniken, Karolinska sjukhuset

Stockholm Sweden

Ethics approval and approval from the Medical products agency Dnr 02-410. 2003-02-03.

Protocol Date

November 2004

Principal investigators signature

**2. Project summary**

**2.1. Justification of the project**

Uterine fibroids are a benign but common condition among women in reproductive age. It is one of the most common reasons for hysterectomy since it often causes bleeding problems sometimes leading to anemia. Several alternative treatment regimens have been investigated that could replace surgery. The antiprogesteron, mifepristone, is one of the most promising drugs that have been tested. In addition to the inhibiting effect on the growth of uterine fibroids antiprogestins have been proposed to have an antiproliferative effect on breast tissue.

**2.2. Proposed research**

The purpose of the present study is to evaluate the effect of mifepristone on the volume of uterine fibroids. The study will also address the effect of mifepristone on the breast tissue

**2.3. New features**

An evaluation of the effect of mifepristone on uterine fibroids will give information on whether it is possible to treat uterine fibroids pharmacologically instead of using surgery. If that is the case a large number of hysterctomies could be avoided. It would also be of importance to investigate the proposed antiproliferative effect on breast tissue.

**2.4. Techniques and skills**

The study requires experience in ultrasound technique, experience in cytological fine-needle aspiration technique of the breast and evaluation of the biopsies.

**2.5. Problems anticipated**

Timing of the hysterectomy after the treatment period

**3. Description of the project**

**3.1. Rationale and objectives of the study**

**3.1.1. Rationale**

Uterine leiomyomata are common pelvic tumors occurring in up to 20% of women over 30 years of age, and represent one of the most frequent indications for operative procedures in women of reproductive age. (Buttram et al 1981) The growth of the fibroids seems to be dependent of ovarian steroids and receptors for both estrogen and progesterone have been identified in fibroids (Wilson et al 1980, Soules et al 1982).

That progesterone may play a role in leiomyomata growth is further supported by the finding of a higher mitotic count in leiomyomata obtained during the secretory phase than in the proliferative phase of the menstrual cycle. (Kawaguchi et al 1989)

The antiprogesteron mifepristone is a synthetic steroid hormone that binds with high affinity to the progesterone receptor thereby preventing the effect of progesterone. Administration of mifepristone has various effects on reproductive function, depending on the dose and stage of the menstrual cycle. When administered in the follicular phase of the cycle, mifepristone inhibits the process of follicular maturation and blocks ovulation.

Previous studies have shown positive effects after treatment with mifepristone when administered 25-50 mg daily for 3 months. All women became amenorrhoeic during treatment and the size of the fibroids decreased with approximately 50% (Kettel et al 1994). Studies using even higher doses of mifepristone for longer periods than 3 months have not revealed any significant risk of endometrial hyperplasia (Murphy et al 1995).

Ovarian steroids also have an effect on breast tissue. Concern has been raised about hormone replace treatment (HRT) with estrogen and progesterone. Various antiprogestins have been proposed as effective for inhibiting tissue growth in the breast. It would therefore be of importance to evaluate the effect of antiprogestins on the breast tissue.

Apoptosis (programmed cell-death) and cell proliferation seem to be closely linked according to experimental evidence. Many proteins that can induce cell death are also components of the cell cycle (Meikrantz et al 1995, Huang et al 1997). The balance between cell proliferation and cell death determines tumor growth in the endometrium as well as in the breast tissue. Specific markers for proliferation (Ki-67) and apoptosis (proteins from the Bcl-2- family) have been identified.

If a non-surgical method could be found in the treatment of uterine fibroids it might benefit both the society in forms of reduced cost but most of all, the individual woman. From the preliminary data in vitro it seems possible that antiprogestins could prevent growth or even inhibit development of breast cancer.

**3.1.2. Objectives**

The objectives of the present study are

1. To develop a new non-surgical method for treatment of uterine fibroids
2. To study the effect of mifepristone on the size of uterine fibroids
3. To evaluate factors regulating fibroid growth by comparison with untreated fibroids and normal myometrium
4. To study the effect of mifepristone on the amount of blood loss and pelvic pain in patients with fibroids
5. To study the effect of mifepristone on proliferation of breast tissue
6. To study the effect of mifepristone on body composition and cortisol levels

**3.2. Previous** **similar studies**

Results from previous relevant studies have been described above

**3.3. Design and methodology**

**3.3.1. General outline**

This is a pilot study to evaluate the effect of mifepristone on uterine fibroids. The study will include 40 women with uterine fibroids, recruited from the waiting list for hysterectomy. After inclusion women will be randomized to treatment with mifepristone or a placebo (i.e B-vitamin tablets). An ultrasound examination will be performed at start and repeated every 4^th^ week. The total volume of the uterus will be measured as well as each fibroid, and also a Doppler examination of the blood vessels to the fibroids. An biopsy from the fibroid will be obtained at the same time. Endomtrial biopsies will be obtained prior to study start and at surgery for safety reasons and will undergo histological analyses.

Blood will be drawn for analyses of liver function and hemoglobin values as well as estradiol, progesterone, LH, FSH, SHBG, androstendione and DHEAS once a month. A 24 hour urine collection for cortisol levels will be performed and a measurement of body composition with bioelectrical impedance analysis (BIA). This will give information about body cell mass, fat-free mass and total body water. These tests will be repeated after the treatment period.

Women that have not had a mammography during the last 12 months will be offered to have one before the start of the study. A fine-needle-aspiration, from one breast will be performed before, and after the treatment period, for cytological analyses of apoptotic and proliferative factors i.e. Bcl-2, Ki-67 and receptors for estrogen and progesteron. Fine needle aspirations will be performed at the Dept of Cytology, KS in collaboration with Professor Lambert Skoog.

The women will be given Mifegyne 200 mg tablets or B-vitamins and instructed to divide the tablets into 4 parts. One quarter of the vitamin or Mifegyne tablet (50 mg mifepristone), will be taken orally every other day for 3 months. A diary card will be handed out for self registration of bleeding (days and amount), pelvic pain and any side effects.

After the treatment period the woman will undergo surgery as planned. After the uterus has been removed it will be weighed and biopsies from the myomas will be taken for analysis of apoptotic and proliferative factors i.e. Bcl-2, Ki-67 and steroid receptors. Finally the uterus will be examined by a pathologist as part of the usual routine procedure.

**3.3.2. Criteria for selection of subjects**

A total of 40 women will be recruited among women that are already on the waiting list for hysterectomy.

Inclusion criteria:

- Uterine fibroids requiring treatment
- Good general health
- Willing and able to participate after giving informed consent

Exclusion criteria

- Need for immediate surgery
- Concomitant hormonal treatment (HRT)
- History of malignant disorder of the breast
- Any contraindication for mifepristone

Criteria for retrospective exclusion

Subjects may be excluded from analysis if one of the following applies:

- Any violation of the study protocol
- Lack of essential data

**3.3.3. Subject allocation**

The women who are willing to participate and fulfill the inclusion criteria will be randomized to treatment with Mifegyne or B-vitamin (control group). The treatment will be double blind. Only the nurse instructing patients on how to divide the tablets will be aware of treatment group. The women will obtain once monthly 6 tablets of 200 mg mifepristone or 6 tablets of B-vitamin of which one quarter of a tablet will be taken every other day.

**3.3.4. Description of the drug to be studied.**

Mifepristone

1. Chemical name: 17beta-hydroxy-11beta-[p-(dimethylamino)-phenyl]-17-(1-propynyl)estra-4,9-dien-3-one
2. Chemical structure:

1. Empirical formula: C_29_H_35_N_1_O _2_
2. Route of administration: oral tablets
3. Amount present per tablet: 200 mg (divided into 4 parts of approximately 50mg)

**3.3.5. Admission procedure**

Eligible patients admitted for hysterectomy will be admitted to the study after they have given their informed consent. Forty subjects will be recruited. The following information will be recorded:

-general and gynecological history

-details of normal cycle (duration of cycle and of menstrual bleeding

-height and weight

-a pelvic examination will be done

**3.3.6. Duration of the project**

It is estimated that 22 months will be required for recruiting the subjects and performing the examinations and 2 months for data analysis.

**3.3.8-10 Data management and analysis**

# All data will be recorded in the patients file. The analysis of data will be done at the center

## Laboratory analysis

# Hormone analyses will be preformed at the department of Clinical Chemistry.

# Breast tissue will be analysed in collaboration with Professor Lambert Skoog. KS.

Myometrial bioses will be analysed together with Associate Professor Matts Olovsson KK, UAS. Part of the biopsies from the uterine fibroids and myometrium will be snap frozen and stored in liquid nitrigen until further processed. Material will undergo receptor analysis, immunohistochemistry and in situ hybridization. Part of the biospies will be embedded in paraffin and used for immunohistochemistry. Microarrays will be used to study gene expression of relevant growth factors.

## Statistics

*Sample size calculation*Assuming a standard deviation of 10% in the percentage volume change, 18
 subjects per group will be required to detect a difference of at least 10%
 in the percentage volume change between the treated and the placebo groups
 with 90% power, using a one-sided 5% level test. Allowing for a 10%
 drop outs, 20 subjects per group, or a total of 40, will be recruited into
 the trial.

*Analysis*

 Myoma volume: Repeated measures analysis techniques will be used. For each group, the
 change at each time point will be compared with the baseline value and the
 percentage change with respect to baseline will be estimated. A repeated
 measures model with the baseline volume as a covariate will be used to
 compare the volume at different times between the two groups. Changes will
 be plotted on the absolute scale and will also be expressed as percentage
 change with respect to baseline.

# Bleeding pattern, BIA and laboratory analysis will be compared using the students-t-test.

For evaluation of immunohistochemistry and molecular biology assays of uterine and breast tissue the paired and unpaired Wilcoxon´s tests will be used as appropriate.

**3.4. Project management**

The project will be conducted at the Stockholm WHO centre for Research in Human Reproduction, Karolinska Hospital.

**3.5. Links with other projects.**

None

**3.6. Main problems anticipated**

The principal investigator has vast experience in ultrasound examination technique and the centre has also a well equipped laboratory for the experimental work concerning the myometrial tissue. Some of these analyses will be perfomed in collaboration with associate professor Matts Olovsson, Dept. of Obstetrics and Gynecology, UAS, Uppsala. The fine-needle aspiration for cytology will be performed and analyzed in a near-by laboratory by skilled physicians. Analyses will be performed in collaboration with Professor Lambert Skoog

Since 50 mg Mifepristone tablets are not available in Sweden, 200 mg tablets will be divided into 4 pieces containing approximately 50 mg each. An possible uneven active drug content in the 4 pieces will have no practical implication since doses ranging from 12.5 mg of mifepristone has previously been shown to be effective. Doses much higher than 25 mg have been shown not to be harmful. The long half life of mifepristone is probably also of importance to make a every-second-day approach possible. This approach has previously been successfully used in a clinical study.

Mifegyne placebo tablets are not available from Exelgyn. B-vitamin tablets will therefore be used instead of placebo. The B-vitamin tablets will not influence the studied parameters.

**3.7. Expected study outcome**

Results from this study will increase the knowledge on medical alternatives in leiomyomata treatment and also on the antiprogestagen effect on breast proliferation.

**3.8. References**

Wilson EA, Yang F, Rees E. Estradiol and progesterone binding in uterine leiomyomata and in normal uterine tissues. Obstet Gynecol. (1980) 55:20-24

Soules MR, McCarthy KS. Leiomyomatas: steroid receptor content Am J Obstet Gynecol. (1982) 143:6-11

Meikrantz W, Schlegel R. Apoptosis and the cell cycle. J. Cell Biochem. (1995) 58:160-174

Huang DC, O´Reilly LA, Strasser A, Cory S. The anti-apoptosis function of Bcl-2 can be genetically separated from its inhibitory effect on cell cycle entry. EMBO J. (1997) 16:4628-4638

Buttram Jr VC, Reiter RC. Uterine leiomyomata: etiology, symptomatology, and management. Fertil Steril (1981) 36:433-445

Kawaguchi K, Fujii S, Konishi I, Nanbu Y, Nonogaki H, Mori T. Mitotic activity in uterine leiomyomas during the menstrual cycle. Am J Obstet Gynecol (1989) 160:637-641

Kettel LM, Murphy AA, Morales AJ, Yen SS.

Clinical efficacy of the antiprogesterone RU486 in the treatment of endometriosis and uterine fibroids. Hum Reprod. (1994) Jun;9 Suppl 1:116-20.

Murphy AA, Kettel LM, Morales AJ, Roberts V, Parmley T, Yen SS. Endometrial effects of long-term low-dose administration of RU 486. Fertil Steril 1995;63:761-766

**4. Ethical consideration**

Obtaining fine-needle biopsies from the breast has been proved to be a simple and safe procedure. Previous studies have shown that daily treatment with up to 200 mg for a period exceeding 3 months, is safe.

Approval has been obtained from the Karolinska Hospital´s ethical committee

**4.1. Informed consent and confidentiality**

All potential volunteers will be informed about the aims and protocol of the study, expected efficacy of the treatment and the right to withdraw from the study at any time. They will also be informed of the fact that a monitor, not involved in the study will review the data collected. Women participating in the study will be asked to sign a consent form. See appendix (in Swedish)

**4.2. Risk-benefit assessment**

The outcome of this study may give valuable information for the development of non-surgical therapy for women suffering from uterine fibroids. It may also give information on the effect of mifepristone on the breast.

There is no previously described adverse event connected to the proposed procedures.

### Adverse Events

Any unexpected adverse event that occur during the study will be reported to Läkemedelsverket according to LVFS 1996:17. This will also be recorded in the patient file.

The file will be kept for 15 years after end of study.
